# The effect of stress on rates of asexual reproduction in an invasive planarian

**DOI:** 10.1007/s10646-023-02713-z

**Published:** 2023-11-17

**Authors:** Sebastian L. Rock, Zowi Oudendijk, Fabian T. Kürten, Leonardo Veglia, Valentina Tyukosova, Ioanna Bourtzi, Nicholas Verzé, John J. Sloggett

**Affiliations:** 1 Faculty of Health, Science and Technology, Department of Environmental and Life Sciences, Biology, Karlstad University, Karlstad, Sweden; 2 Maastricht Science Programme, Maastricht University, Maastricht, The Netherlands; 3 Organismal and Evolutionary Biology Research Program, Faculty of Biological and Environmental Sciences, University of Helsinki, Helsinki, Finland

**Keywords:** Asexual reproduction, *Girardia tigrina*, Invasive species, Stress, Flatworm

## Abstract

Animal reproduction under stressful conditions is often reduced, with current survival and future reproduction being generally traded off against current reproductive activity. This study examines the impacts of physical and chemical stressors on the rates of asexual reproduction of the invasive planarian *Girardia tigrina*. 320 wild-caught planaria (mixed size class) were kept individually in Petri dishes such that their individual rates of fission through fragmentation could be easily monitored. Four treatment groups were compared, one chemical (5 mg/L ammonia) and one physical (decapitation), in comparison to a negative control (animals were starved of food) and a positive control where the animals were given an abundance of food. The two treatment groups immediately began reproducing asexually and accumulated the highest number of fissions over the course of the 12-day investigation period, while the positive control only began to fission after 7 days. We propose that the reproductive response observed here is an adaptive one to stressful conditions, whereby the likelihood of survival through numerical abundance is enhanced, although the size and vulnerability of resulting fragments may impose a balancing cost. The response may play a role in the invasiveness of *G. tigrina* by making it able to colonize environments where adverse conditions prevail.

## Introduction

The proximate and ultimate interactions between environmental stress and reproduction are significant in a wide diversity of contexts, including human and animal health, agriculture and species conservation (Schreck et al. 2001; Amat et al. 2016; Parajuli et al. 2019; Valsamakis et al. 2019). In animals, sexual reproduction under stressful conditions is often retarded, with current survival and future reproduction being traded off against current reproductive activity (Fielenbach and Antebi 2008; Schoech et al. 2009), thus, reproductive activity decreases. Normally, asexual or parthenogenetic animals show a directly induced decrease in reproductive rate as a response to environmental stress (Parish and Bale 1993; Haridevan et al. 2015; Zheng et al. 2017). Even in taxa with both sexual and asexual forms, sexual reproduction commonly replaces asexual reproduction under high stress conditions (Morran et al. 2009; Crummett et al. 2013). Rather few cases are documented where an increased reproductive rate is observed in an asexual animal in response to stress and still less where this is likely to be adaptive (Piranio et al. 1996). Aphids may increase their reproductive rate in response to moderate water stress in their host plants; however, this could be due to changes in plant nutrient availability to the aphids (Banfield-Zanin and Leather 2015); similarly budding in the sponge *Cinachyrella cavernosa* increases with temperature, interpreted as a stress response (Singh and Thakur 2015); however, within limits, as all ectotherms are known to increase activity with rising temperature.

Many phyla reproduce asexually, including annelids (Gibson and Harvey 2000; Kostyuchenko et al. 2016), echinoderms (Jaeckle 1994; Rubilar et al. 2005), cnidarians (Bell and Wolfe 1985; Lucas 2001), platyhelminthes (Saló 2006; Egger et al. 2007) and arthropods (Banfield-Zanin and Leather 2015; Gutekunst et al. 2018). Planarian asexual reproduction has been a well-documented phenomenon since the 1700s when well-known researchers such as Michael Faraday first recorded it (Hirshfeld 2006). Planarian asexual reproduction takes several forms including parthenogenesis and fission by fragmentation (Lentati 1970; Åkesson et al. 2001; Egger et al. 2007; Malinowski et al. 2017). Planarian fission by fragmentation is a product of a three-step process: waist formation, pulsation, and rupture. Waist formation narrows a portion of the body of an animal (typically behind the pharynx) thus allowing for increased localized strain. Pulsation then pulls the two ends of the animal apart increasing strain on the waist, until the body ruptures at roughly the waist, thus concluding the fission process (Malinowski et al. 2017). This process generates two sections of planarian tissue which will then regenerate the organs not already present in that tissue section, and thus two new planarians. This is made possible by the animal’s incredibly well developed regenerative capacity.

*Girardia tigrina* (Girard) displays both sexual and asexual reproduction, with the majority of European populations being obligately asexual through fission by fragmentation (Benazzi 1993). This North American planarian can be found in a wide array of freshwater ecosystems (Reynoldson and Young 2000) and is highly invasive, having colonized Europe over the course of the 20th century, and, more recently Japan and Morocco (Reynoldson 1956; Gourbault 1969; Sluys et al. 2010; Alonso and Camargo 2015; Kanana and Riutort 2019; Mabrouki et al. 2023). The invasion of *G. tigrina* is likely facilitated by ecological factors, such as greater foraging ability when compared to native species (Pickavance 1971a, b; Gee and Young 1993); however, numerical abundance as a product of asexual reproduction could also facilitate this. Motivated by our interest in the invasiveness of this species, we here examine the link between rates of asexual reproduction and stress in *G. tigrina*. We elected to compare the impacts of chronic ammonia toxicity, an ecologically relevant and previously studied chemical stressor (Alonso and Camargo 2015) to the physical injury of the animal (decapitation, easily repeatable and well studied). The effects of these two treatment groups were controlled for by both positive and negative controls: well-fed animals are known to increase their asexual reproduction, while an unfed group controlled for the starvation stress experienced by the animals in the other treatments.

## Materials and methods

### Animal material

**Fig. 1:**
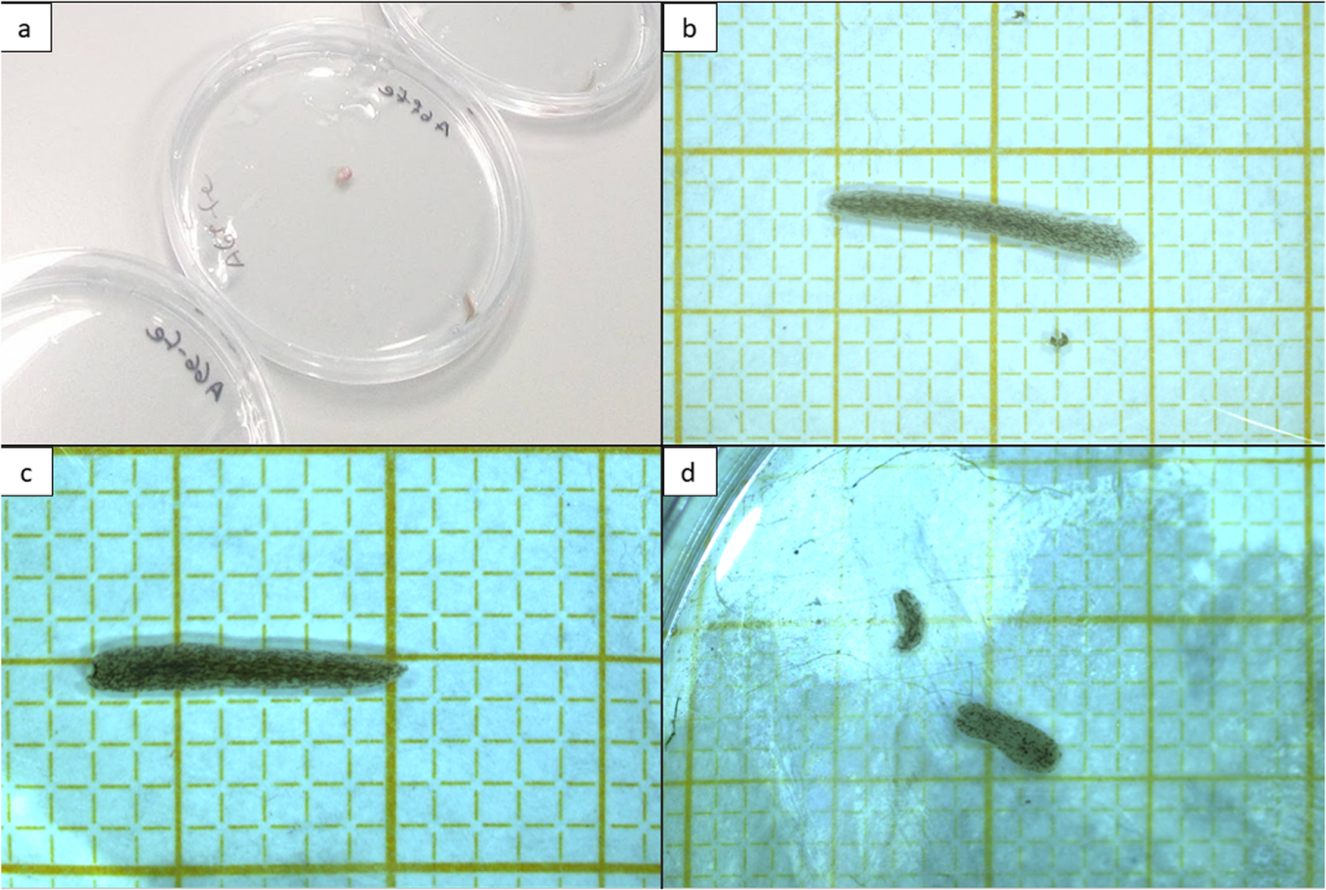
Experimental setup for all treatment groups (**a**). Examples of intact (**b**), freshly decapitated (**c**) and recently fissioned (d) *G. tigrina*. Distance between thin lines in (**b**–**d**) represent 1 mm

*G. tigrina* specimens were collected from an invasive population of the Jeker River in Maastricht, The Netherlands (50.8438N, 5.6861E) in early-June 2017. The flatworms were collected off the bottom of rocks with soft paint brushes. When brought to the laboratory, the planarians were housed individually in 90 mm Petri dishes and maintained in a climate-controlled cabinet at 15 °C (same as river) with 85% humidity to reduce evaporation, and no light since, like many planarians, *G. tigrina* is photophobic (Hinrichsen et al. 2019; Fig. 1). The flatworms were exposed to a minimum of two hours of light per day during the regular observation and maintenance procedure, an important note as perpetual darkness increases the rate at which *G. tigrina* fissions (Morita and Best 1984). The same conditions were used throughout the experimental process. The Petri dishes were filled with non-carbonated bottled mineral water (Albert Heijn natural mineral water). Animals were acclimated to lab conditions two weeks before the start of the experiment, four days before the experiment the planarians were fed earthworm segments [*Dendrobaena veneta* (Rosa)] from worms that had been stored in a glass beaker with moist paper towels for three days. The storage period allowed for the majority of intestinal matter, containing potentially noxious material, to be egested by the earthworms. Asexuality of this population was determined after dissecting some individuals and not observing any reproductive organs. Moreover, the authors have previously kept this population captive for extended periods, under various conditions, no egg cocoons were ever observed.

### Experimental process

**Fig. 2:**
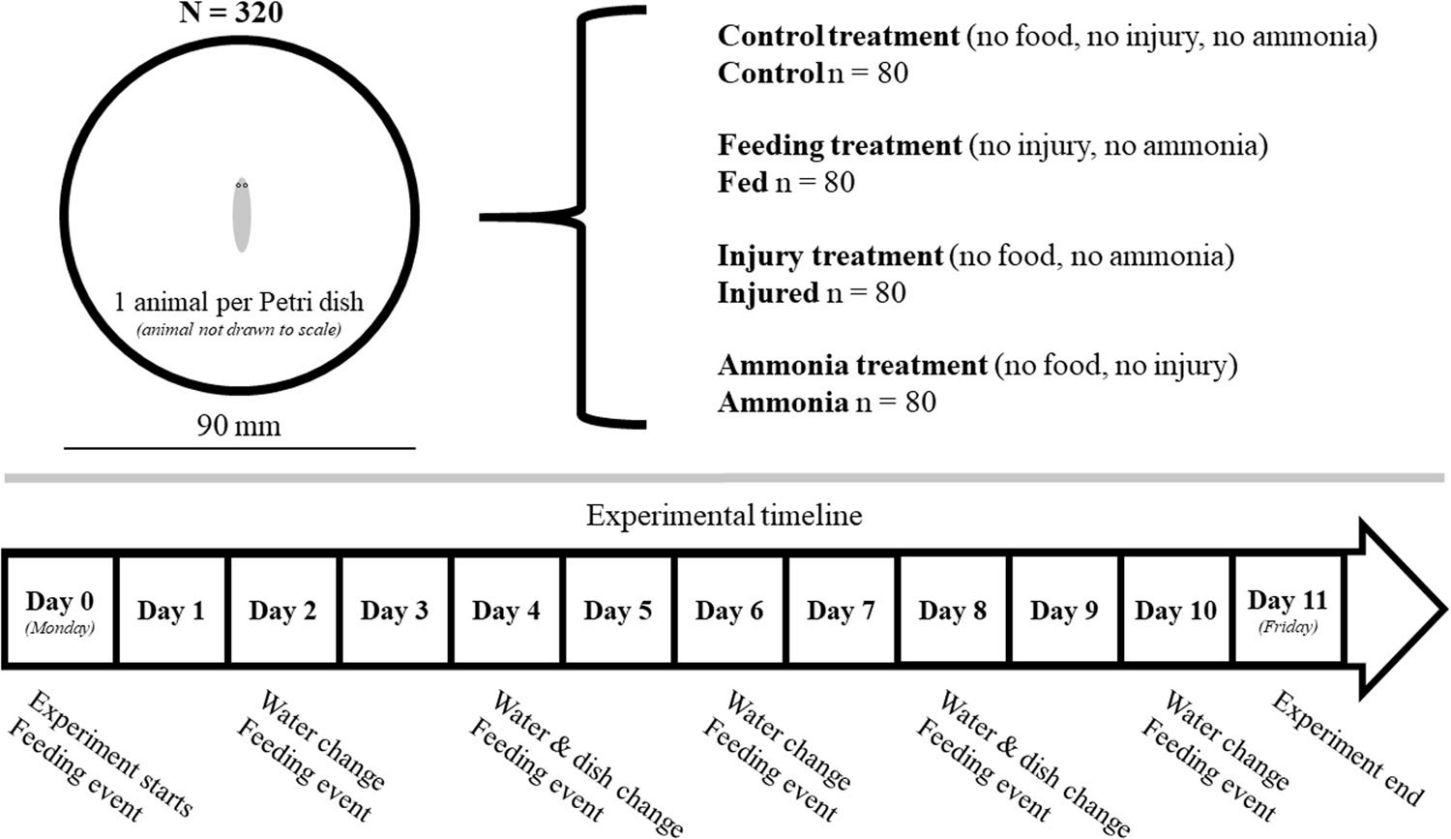
Graphical method of the experimental setup, with an accompanying timeline of manipulations

The planaria (*N* = 320; mixed size class) were tested under three treatments and a control: the first treatment (Ammonia), where the animals were chronically exposed to sub-lethal levels of ammonia (5 mg/L), the second treatment (Injured), where the animals were injured via decapitation, the third treatment (Fed), where the animals were fed every other day, and the unpolluted, uninjured, unfed control maintained in only mineral water (Control; Fig. 2). The first two conditions represented two distinct forms of stress (the ammonia condition being chemical and decapitated being physical) and the third representing improved conditions over the control and acted as a positive control. All planarians were fasted for the duration of the experiment, with the exception of Fed treatment. As decapitated planarians do not show interest in food for several days (Shomrat and Levin 2013), and can survive for long periods without feeding (Newmark and Alvarado 2002), fasting all animals prevented differences in nutritional status confounding the results. Flatworm size was not recorded, but controlled for through sheer numerical abundance (starting length 8–21 mm; *n* = 80).

The concentration of 5 mg/L ammonia is naturally occurring and, in this population, sublethal to *G. tigrina*. currently unpublished results show 35% mortality at 15 mg/L after 14 days of chronic exposure for decapitated individuals (Tolkamp 1990; Rock et al. 2017, *unpublished data*). To treat the animals with ammonia, a 500 mg/L stock solution was prepared with ammonium chloride. When the water was changed on the ammonia treatment, the previously prepared stock solution was used diluted to reach 5 mg/L, then used for the water change rather than clean water. Injured flatworms were decapitated with a razor blade below the sensory lobes and heads removed from the dish (Fig. 2). The Fed group was allowed to feed on a section of *D. veneta* roughly three segments long, maintained as previously described, for one hour before the water change. For all treatments, water was changed every other day to maintain cleanliness as well as to ensure a constant level of ammonia in that treatment group. The Petri dishes were changed every four days. The experiment ran for 11 days, during which all instances of fission were recorded daily. When one animal fissioned more than once, each instance was counted independently. This allowed for a comparative analysis of the total number of fissions over time per condition, along with an investigation into the number of individuals that fissioned. Fissioned sections were left in the Petri dishes, no mortality was observed over the course of this study.

### Statistical analysis

Chi-squared tests for heterogeneity were used to investigate differences in fission behavior between the four groups; these compared the number of overall fissions and the number of animals that fissioned. As a post-hoc analysis, Mann-Whitney *U* tests were run between treatments. The significance level was adjusted with a Bonferroni correction to account for multiple comparisons (six tests; significance level = 0.0084). A Fisher’s exact test was used to compare the proportions of multi-fissioning planaria to single-fissioning planaria between treatment groups. All analysis was performed with SPSS V28.

## Results

**Fig. 3:**
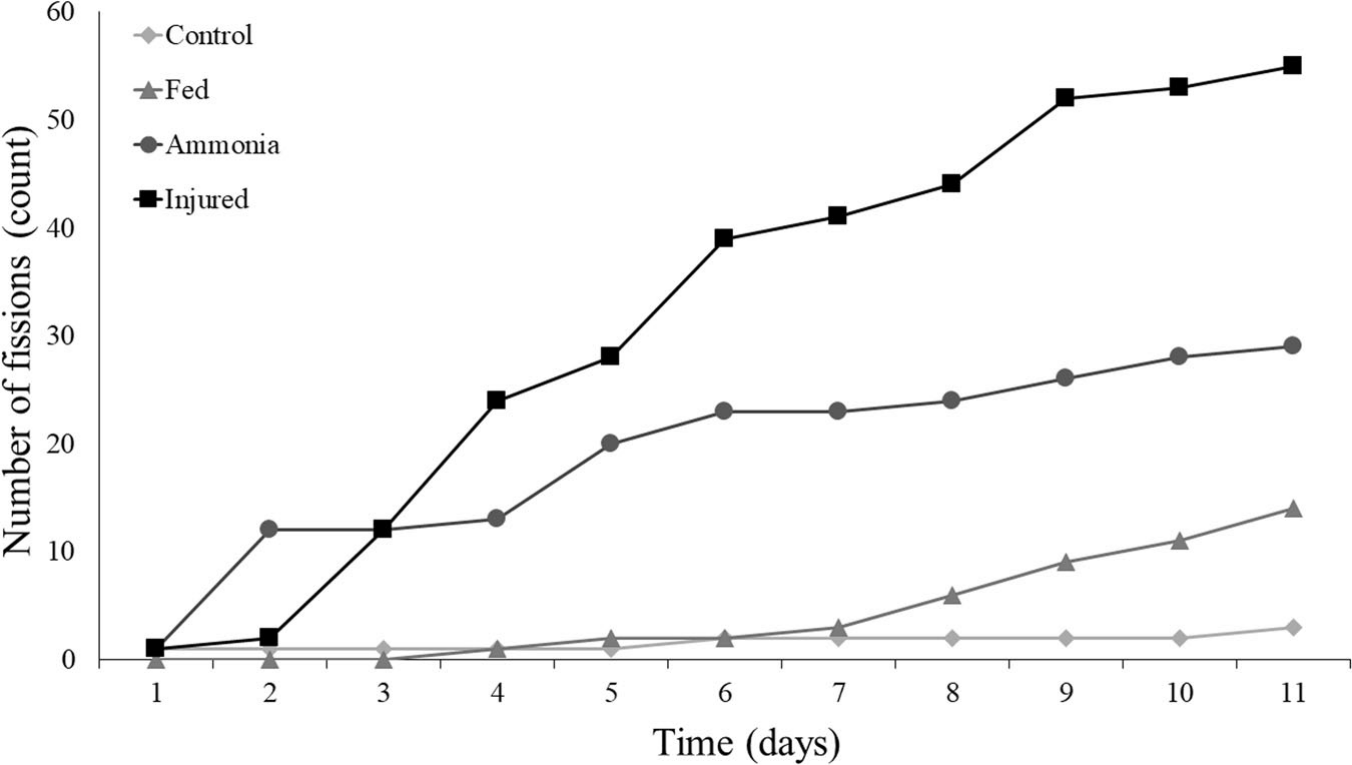
Total number of fission incidents by *G. tigrina* for each condition over the 11 days of the experiment

Significant heterogeneity was recorded between the number of fissions in the different treatment groups (*X*^2^ = 66.309, *df* = 9, *p* < 0.0001). By the study endpoint, the injured group accumulated the highest number of fissions, followed thereafter by the ammonia treatment. The fed group initially remained identical to the control group but began to accumulate fissions after 7 days (Fig. 3). After 11 days, all treatment groups were significantly different from each other, with the exception of the ammonia and fed groups, which did not reach the adjusted significance level (*p* = 0.0322; Table 1).

**Table 1:** Mann-Whitney U test results comparing the total number of fissions accumulated by *G. tigrina* across treatment groups

Group comparison	N	*U* - score	*Z* - score	Sig.
Control vs Fed	160	2798.5	−2.636	*0.0084*
Control vs Ammonia	160	2352.5	−4.447	*<0.0001*
Control vs Injured	160	1508.0	−7.210	*<0.0001*
Fed vs Ammonia	160	2739.5	−2.142	0.0322
Fed vs Injured	160	1889.5	−5.300	*<0.0001*
Ammonia vs Injured	160	2371.5	−3.216	*0.0013*

Results significant under the Bonferroni corrected level (*p* < 0.0084) are reported in *italics*

**Fig. 4:**
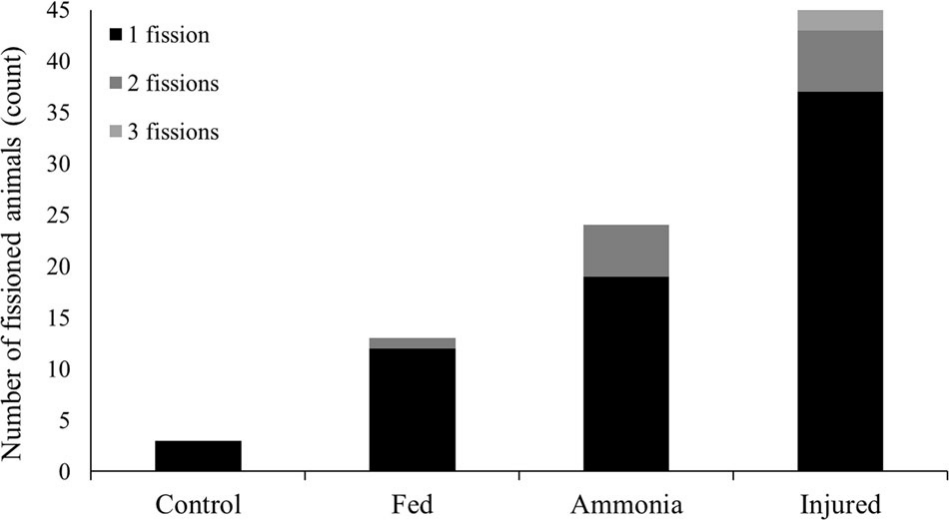
Number of *G. tigrina* individuals which fissioned one, or multiple times by the end of the 11 days investigation (n = 80; N = 320)

The differences in total fissions between the groups was a result of significant heterogeneity between the number of individuals that fissioned within each treatment group (*X*^2^ = 62.334, *df* = 3, *p* < 0.0001). This followed the same pattern as before, where the injured treatment induced the highest number of planaria to fission, while the negative control treatment induced the fewest (Fig. 4). All treatment groups appeared significantly different from each other, with the exception of the fed group which was not statistically different from either the control group (*p* = 0.0398) or the ammonia group (*p* = 0.0086; Table 2) and the adjusted significance level.

**Table 2:** Mann-Whitney U test results comparing the total number of fissioned *G. tigrina* across treatments groups

Group comparison	N	*U* - score	*Z* - score	Sig.
Control vs Fed	160	2800.0	−2.627	0.0086
Control vs Ammonia	160	2360.0	−4.419	*<0.0001*
Control vs Injured	160	1520.0	−7.223	*<0.0001*
Fed vs Ammonia	160	2760.0	−2.056	0.0398
Fed vs Injured	160	1920.0	−5.246	*<0.0001*
Ammonia vs Injured	160	2360.0	−3.342	*0.0008*

Results significant under the Bonferroni corrected level (*p* < 0.0084) are reported in *italics*

As the number of planaria that fissioned increased, so did the number of planaria that fissioned multiple times (Fig. 4). The control group had no animals fission more than once, while the injured group had two instances of a planarian fissioning three times. The results of the Fisher-Freeman-Halton Exact Test (*p* = 0.775) do not indicate a significant association between the treatment group and the number of animals that fissioned multiple times.

## Discussion

The results of this study provide an intriguing insight into the relationship between asexual reproduction and the environment in *G. tigrina*, which may shed some light on this species invasiveness. The results of this study could benefit from further extension such as to other types of injury (e.g. incisions/holes), other potential stressors (e.g. other pollutants/salinity/light) as well as a longer experimental timeframe.

The stress conditions were chosen as samples of natural and realistic stressors. Many freshwater habits are eutrophic because of anthropogenic activity, therefore, *G. tigrina* can be exposed to high nitrogen levels. The concentration of ammonia used here was one third of that known to show mortality in *G. tigrina*, and has been shown to be toxic to some native Dutch flatworm species (Rock et al. 2017, *unpublished data*). As a result of their ability to regenerate, planarians are able to survive serious injury from predators; such as decapitation, which normally leads to death in most other taxa.

There are several known variables that determine the rate at which planarians fission, such as size of the animal, population density, and photoperiod (Child 1910; Best et al. 1969; Pigon et al. 1974; Morita and Best 1984). The higher rate of fission in the Fed group as opposed to the unfed control groups is likely a direct consequence of size, mediated by food availability. Flatworm growth was not recorded in this study, however the Fed group roughly doubled in size, if not more, over the course of the 11 days (*personal observation*). As the animals grew, they began to fission more rapidly. An effect that was seen in the second half of the experiment. Given that *G. tigrina* is successfully able to outcompete native planarian species in foraging ability, their broader resource spectrum likely allows the species to grow faster, and thus reproduce more when compared to the native varieties; a key factor in their invasive success (Pickavance 1971a, b).

The planarians in both of the stress conditions in this study increased their rate of fission over the control despite neither having an obvious direct effect. While the ammonia treatment increases allosteric strain on the animals, increasing the energy required for basic body maintenance, injury physically removes nutrient from the body. We, therefore, tentatively suggest that the animal is exhibiting an adaptive indirect response to stress. Asexual organisms can reach high population densities (Simon et al. 2010) and lineages can potentially persist through their sheer numerical abundance. Fission, in this case, could serve to accentuate the properties of asexual reproduction, i.e., a planarian could increase the likelihood of future survival through the production of as many clones as possible via the process of fission.

The heightened level of ammonia constitutes an existential threat to all aquatic organisms. Ammonia levels may fluctuate in water bodies over space and time (McColl 1974; Wurts 2003), and has been reported toxic to aquatic organisms from levels as low as 0.53 mg/L (EPA, USA 2013). A strategy to produce numerous clonal offspring would therefore increase the likelihood of survival through an adverse period. The level of ammonia chosen here did not result in any mortality to the animals in this study and was within natural levels for the river and previously defined toxic concentrations (Tolkamp 1990; EPA, USA 2013, Rock et al. 2017).

Injury to the animals via decapitation, on the other hand, has been previously shown to increase the rate of fission (Brondsted 1955; Morita and Best 1984; Hori and Kishida 1998), although such experiments have been focused on proximate physiological mechanisms rather than ultimate ones (Morita and Best 1984; Hori and Kishida 1998). As rates of fission also increase when the animals are in perpetual darkness, it has been proposed that these are mediated by the same mechanism, as with no head, there are no eyes to perceive light (Asano et al. 1998). We generally avoided this photoperiod effect by exposing the animals to light for at least two hours a day for maintenance and observation purposes, which is sufficient to abolish this effect (Morita and Best 1984). An argument could be made that the increased fission observed in the injured group was a result of the photoperiod effect rather than the injury itself. However, decapitated *G. tigrina* regenerates eyespots after 3 days of regeneration (López et al. 2019), which would have limited the effect to the first half of the study. As full head regeneration is generally assumed to take at least 6 days (Cebrià et al. 1997), the increase in fission could be mediated by some other structure not yet regenerated.

It is clear both in the case of ammonia and decapitation, that the products of fission are initially much smaller than the original planarian; they are also typically missing eyes and other essential organs, and are unable to feed. All these factors potentially act to reduce their fitness. The fissioning strategy would therefore have to offset these costs. Since the number of individuals is doubled by splitting into two, these costs would need to be relatively high. In light of the risk imposed, further research should examine the relative benefits of an increased rate of reproduction relative to the costs of small size and temporary loss of essential body parts, in addition to expanding this work to a wider range of stressors. If fission is adaptive this may help explain the invasiveness of *G. tigrina* relative to other flatworm species. The success with which this species has been able to invade new territory could be a consequence of anthropogenic effects on freshwater ecosystems, such as pollution, which increases stress on the organisms that live there. Future investigations should be conducted to confirm or refute this hypothesis.
